# Salt forms of the monoazo dye Mordant Orange 1

**DOI:** 10.1107/S2053229626006765

**Published:** 2026-07-07

**Authors:** Alan R. Kennedy, Connor MacCall, Michael W. Reid, Ben Shaw, Maxwell Taylor

**Affiliations:** a Department of Pure & Applied Chemistry, University of Strathclyde, 295 Cathedral Street, Glasgow, G1 1XL, Scotland, United Kingdom; University of Monash, Australia

**Keywords:** crystal structure, colourant, dye, salt selection, azo, carboxyl­ate ligands, coordination polymer

## Abstract

The crystal structures of eight salt forms of the carboxyl­ate-functionalized monoazo dye Mordant Orange 1 are pre­sent­ed and discussed, primarily in terms of their coordination behaviour. The relatively non-polar metals Mg and Li give the solvent-separated ion-pair structures hexa­aqua­magnesium bis­{2-hy­droxy-5-[(*E*)-(4-nitro­phen­yl)diazen­yl]benzoate} tetra­hydrate, [Mg(H_2_O)_6_](C_13_H_8_N_3_O_5_)_2_·4H_2_O, and tetra­aqua­lithium 2-hy­droxy-5-[(*E*)-(4-nitro­phen­yl)diazen­yl]benzoate dihydrate, [Li(H_2_O)_4_](C_13_H_8_N_3_O_5_)·2H_2_O, respectively. The alkaline earth metals Sr and Ba give isostructural *catena*-poly[[[(di­methyl­form­amide)stron­tium(II)]-μ-aqua-bis{μ-2-hy­droxy-5-[(*E*)-(4-nitro­phenyl)di­azen­yl]ben­zoato}] di­methyl­form­amide di­sol­vate] and the barium(II) analogue, {[*M*(C_13_H_8_N_3_O_5_)_2_(DMF)(H_2_O)]·2DMF}_*n*_ (*M* = Sr, Ba; DMF is di­methyl­formamide, C_3_H_7_NO), and a crystallographic mirror plane passes through the metal centre and the DMF ligand. These are 1D coordination polymers that propagate *via M*—O—*M* bridges where the O atom is from a carboxylate group or from a water ligand. For the Na, Rb and mixed Cs/Na salt forms, namely, poly[tetra-μ-aqua-di­aqua­bis­{μ-2-hy­droxy-5-[(*E*)-(4-nitro­phen­yl)diazen­yl]benzoato}disodium(I)], [Na_2_(C_13_H_8_N_3_O_5_)_2_(H_2_O)_6_]_*n*_, poly[tetra-μ-aqua-bis­{μ-2-hy­droxy-5-[(*E*)-(4-nitro­phen­yl)di­azen­yl]benzoato}dirubidium(I)], [Rb_2_(C_13_H_8_N_3_O_5_)_2_(H_2_O)_4_]_*n*_, and poly[tetra-μ-aqua-di­aqua­bis­{μ-2-hy­droxy-5-[(*E*)-(4-nitro­phen­yl)diazen­yl]benzoato}cae­s­ium(I)sodium(I)], [NaCs(C_13_H_8_N_3_O_5_)_2_(H_2_O)_6_]_*n*_, the nitro substituent was found to be as com­petitive in its ability to bond to metal as the carboxyl­ate group. For Na, this gave a 1D coordination polymer, where Na_4_ units link through the length of the azo anions and *via* bonds to both nitro and carboxyl­ate groups. For both the Rb and the mixed Cs/Na salt forms, higher metal coordination numbers and more extensive bridging inter­actions give 3D coordination polymers.

## Abstract 2

The crystal structures of eight salt forms of the carboxyl­ate-functionalized monoazo dye Mordant Orange 1 are pre­sent­ed and discussed, primarily in terms of their coordination behaviour.

## Introduction

Acidic substituents, such as sulfonic or carb­oxy­lic acid groups, are often added to organic colourants in order to improve aqueous solubility. These functionalities allow for a variety of salt forms of the dye to be created in order to idealize the material properties of the colourant, in much the same way as salt forms of pharmaceutical materials are created and screened during solid form selection (Stahl & Wermuth, 2008 ▸; Christie, 2014 ▸). The counter-ions used are typically *s*-block metal cations and thus the solid-state structures of many so-called organic dyes are in fact best described as metal coordination com­plexes and often as coordination polymers. Systematic structural studies on *s*-block metal salts of sul­fon­ated monoazo dyes and pigments allowed identification of different structural classes (solvent-separated ion pairs, simple com­plexes and higher connectivity com­plexes) and showed that which structural class was adopted depended on the nature of the cation and on the position of the sulfonate group (Kennedy *et al.*, 2001 ▸; Kennedy *et al.*, 2004 ▸; Kennedy *et al.*, 2006 ▸; Kennedy *et al.*, 2012 ▸). Thus, for instance, with *para*-sul­fon­ated azo dyes, only Mg salts gave solvent-separated structures, whilst for *meta*-sul­fon­ated azo dyes, the next less-polar alkali earth metal Ca also did so. Relationships were also iden­tified relating the conformational twist of the dye anions to the layering motifs adopted in the packed structures (Ken­nedy *et al.*, 2009 ▸). Similar systematic studies of salt forms of azo colourants with *R*COO^−^ substituents seem to be lacking. A search of the Cambridge Structural Database (CSD; Groom *et al.*, 2016 ▸) found only 53 structures with car­box­yl­ate-substituted azo­benzene cores and an *s*-block metal counter-ion. However, most of these structures were of azo species with multiple *R*COO^−^ groups and were studied for their inter­est as ligands that may generate MOF (metal–organic framework) type structures rather than for relevance to the dyes industry (*e.g.* Wang *et al.*, 2018 ▸; El Osta *et al.*, 2012 ▸; Meng *et al.*, 2016 ▸). Other examples are structures of pigments containing both sulfonate and carboxyl­ate functions (*e.g.* Beko *et al.*, 2012 ▸; Tapmeyer *et al.*, 2020 ▸; Kennedy *et al.*, 2000 ▸). This leaves only a handful of structures of simple carboxyl­ate azo species that are dye relevant, which is especially surprising for a common functionality with a history of usage going back to the earliest days of chemistry (Christie, 2014 ▸). Herein, we add to this data by presenting the structures of eight salt forms of a simple carboxyl­ated monoazo dye. As well as its more traditional dyestuff roles, Mordant Orange 1 (aka Alizarine Yellow R, colour index number 14030) has been used in the detection of Al^III^ and Cu^II^ (Seleim *et al.*, 2009 ▸), as a pH indicator for relatively basic processes (Martín-del-Río *et al.*, 2013 ▸) and as the colour-change com­ponent in a variety of nanosensors (*e.g.* Ghoniem, 2023 ▸; Rezvani *et al.*, 2023 ▸). Pre­vious crystallographic studies have reported the structures of the free acid form and of the Na^+^ and NMe_4_^+^ salt forms (Yatsenko & Paseshnichenko, 2014 ▸; Yatsenko & Paseshnichenko, 2016 ▸). Newly elucidated here are the structures of the Li, Rb, Mg, Sr, Ba, mixed Na/Cs and NH_2_Me_2_^+^ salt forms, as well as a redetermination of the Na structure. These structures are discussed in terms of their coordination chemistry and are com­pared to their more well-studied sulfonate equivalents.
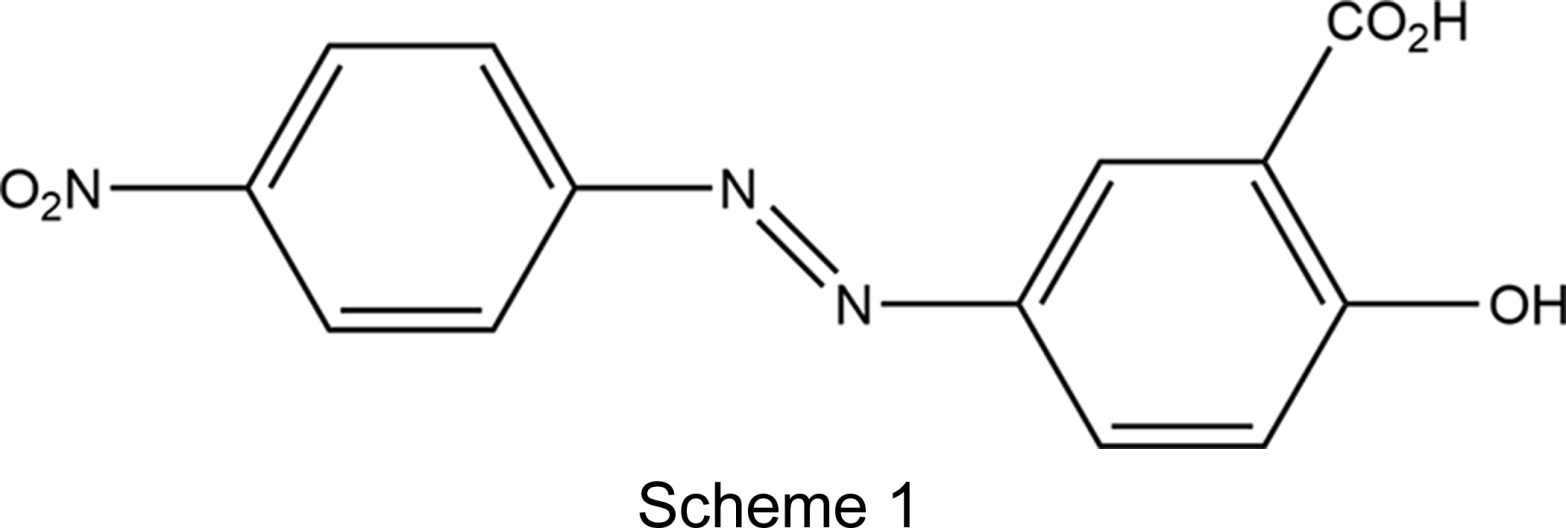


## Experimental

The Na salt of Mordant Orange 1 was purchased from Acros Organics. Crystals were obtained by a simple recrystallization from warm water.

### Synthesis and crystallization

For the CsNa, Mg, Sr and Ba salt forms, a dark-yellow to brown solution was first prepared by addition of approximately 0.1 g (0.32 mmol) of the Na salt to 7 ml of deionized water. To this was added a 5% excess of the appropriate metal chloride. In all cases, slow evaporation of the solvent precipitated orange powders. In the case of the reaction with CsCl, this powder contained crystals of sufficient size and quality for structural determination and gave the CsNa structure re­por­ted. In the case of the Mg salt, suitable crystals were obtained after recrystallization of the initially isolated powder from warm water. The crystals containing Sr and Ba were obtained by recrystallization of the initial products from DMF. Here the powders were dissolved in hot DMF and filtered to give clear solutions. These were left to evaporate slowly, with crystals of the Ba salt appearing after approximately 5 d and of the Sr salt over a period of approximately two weeks.

Using the above conditions with RbCl gave a material which a poor-quality crystal structure identified as a mixed Rb/Na salt, whilst similar experiments with LiNO_3_ gave only the Na starting material. The reported structures were obtained from solutions containing large excesses of the Rb or Li starting material. Thus, for example, 1.0 g (3.2 mmol) of the Na salt was dissolved in 15 ml of warm water. To this was added approximately 2.5 g (36 mmol) of LiNO_3_ in 5 ml of water. Slow evaporation gave crude powders that were recrystallized from warm water to give suitable crystalline samples.

The NH_2_Me_2_^+^ salt form was obtained from the attempted recrystallization of the Na salt from DMF. Initially, no solid products were obtained, but on returning to the samples after approximately 18 months it was found that the solvent had evaporated to dryness leaving behind prismatic red crystals of **[NMe_2_H_2_]MO**.

Multiple attempts to grow suitable single crystals of a Ca salt form failed, despite using a variety of solvents and crystallization methods. Crystals of a K salt form {[K(C_13_H_8_N_3_O_5_)]·2.5H_2_O, *Z*′ = 3, *P*2_1_/*n*; *a*/*b*/*c* = 20.0426 (8)/7.1050 (3)/32.0645 (15) Å and β = 93.038 (4)° at 100 (2) K} were obtained as yellow fibres from aqueous solutions. However, these were twinned, poorly diffracting and gave a final structure with discrepant displacement ellipsoids. There was not enough confidence in this structure for it to be included herein.

### Refinement

Selected crystallographic and refinement parameters for the eight salt forms are given in Table 1 ▸. The structure of the Rb salt was found to be twinned by a 180 ° rotation about [100]. Refinement against a hklf 5 formatted reflection file, generated by *CrysAlis PRO* (Rigaku OD, 2019 ▸), improved both the *R* factors and the residual electron density. The relative contribution of the minor twin com­ponent was refined to 25.29 (10)%. Diffraction spots for the Ba salt form were elon­gated and the indexing of the *b* axis was poorer than ex­pected. This resulted in a somewhat low-grade structure with slightly large residual electron-density features. The isostructural Sr salt did not show these features. The DMF ligands in both the Sr and Ba structures were disordered about crystallographic mirror planes. Appropriate restraints were applied to these groups to ensure that they approximated to normal geometries. In the CsNa structure, the O3*W* water ligand bridges between the Cs and Na centres. A minor-occupancy disordered site for this group (O7*W*) was also refined, corresponding to a terminal water ligand on Cs. As the occupancy of O7*W* refined to just 9.3 (10)%, it has been ignored in the discussion of coordination behaviour below.

H atoms bound to C atoms were placed in idealized positions and were refined in riding modes. The H atoms bound to N atoms and, where possible, those bound to O atoms were refined freely and isotropically. Exceptions were the Rb, CsNa, Mg and Ba salt forms, where all O—H distances were restrained to 0.88 (1) Å, and individual H atoms in the structures of the Li, Na and Sr salt forms were also restrained to have O—H distances of 0.88 (1) Å. Additionally, the H-atom *U^ij^* values in the water mol­ecules were set to be dependant on the *U*_eq_ value of the parent O atom for the CsNa and Ba structures.

## Results and discussion

**Figure 1:**
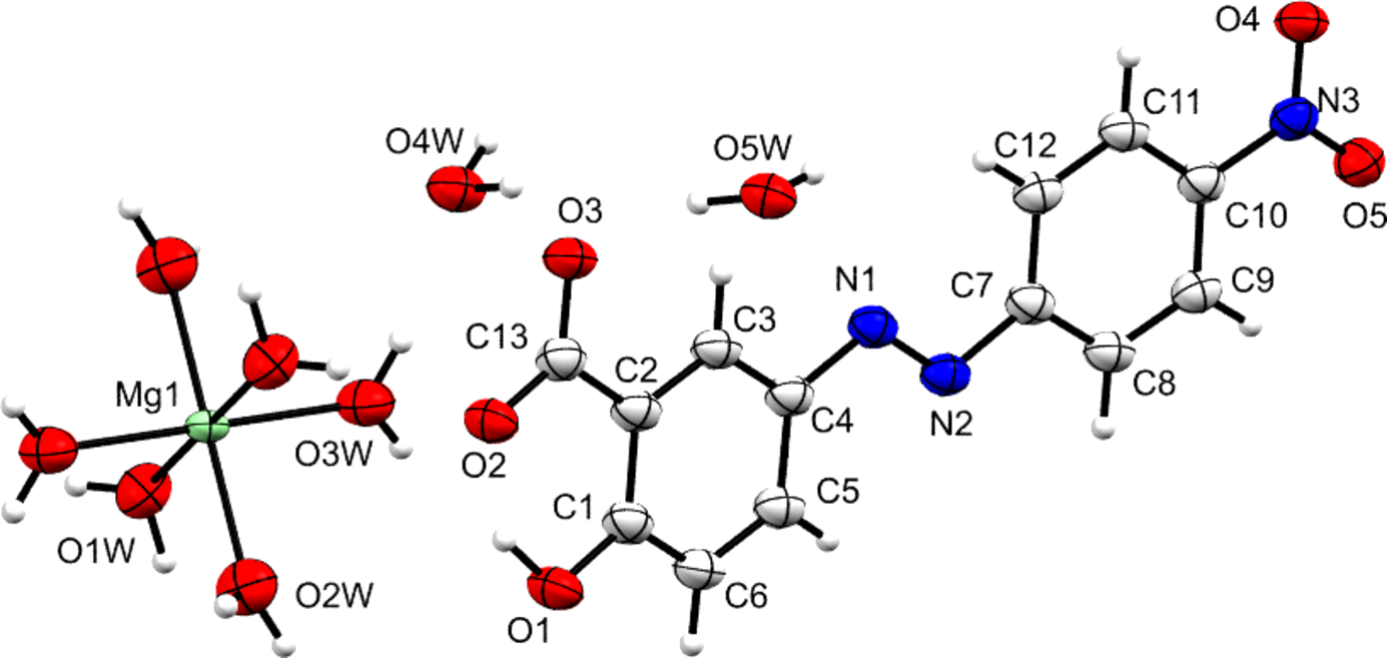
Contents of the asymmetric unit of **MgMO** expanded to show the full coordination of the hexa­aqua­magnesium cation that sits on a crystallographic centre of symmetry. Here and in other diagrams, displacement ellipsoids are drawn at the 50% probability level and H atoms are repre­sent­ed as small spheres or arbitrary size.

A summary of the coordination behaviour of the *s*-block metal salts of **MO** (the C_13_H_8_N_3_O_5_^−^ Mordant Orange 1 anion) is given in Table 2 ▸. Selected bond lengths are given in Tables 3 ▸–7 ▸ ▸ ▸ ▸. Of the seven com­plexes, the coordination behaviour of the Mg and Li salts [Mg(H_2_O)_6_](C_13_H_8_N_3_O_5_)_2_·4H_2_O and [Li(H_2_O)_4_](C_13_H_8_N_3_O_5_)·2H_2_O, hereafter **MgMO** and **LiMO**, respectively, is simplest. These are solvent-separated ion-pair (SSIP) species, as shown in Figs. 1 ▸ and 2 ▸.

**Figure 3:**
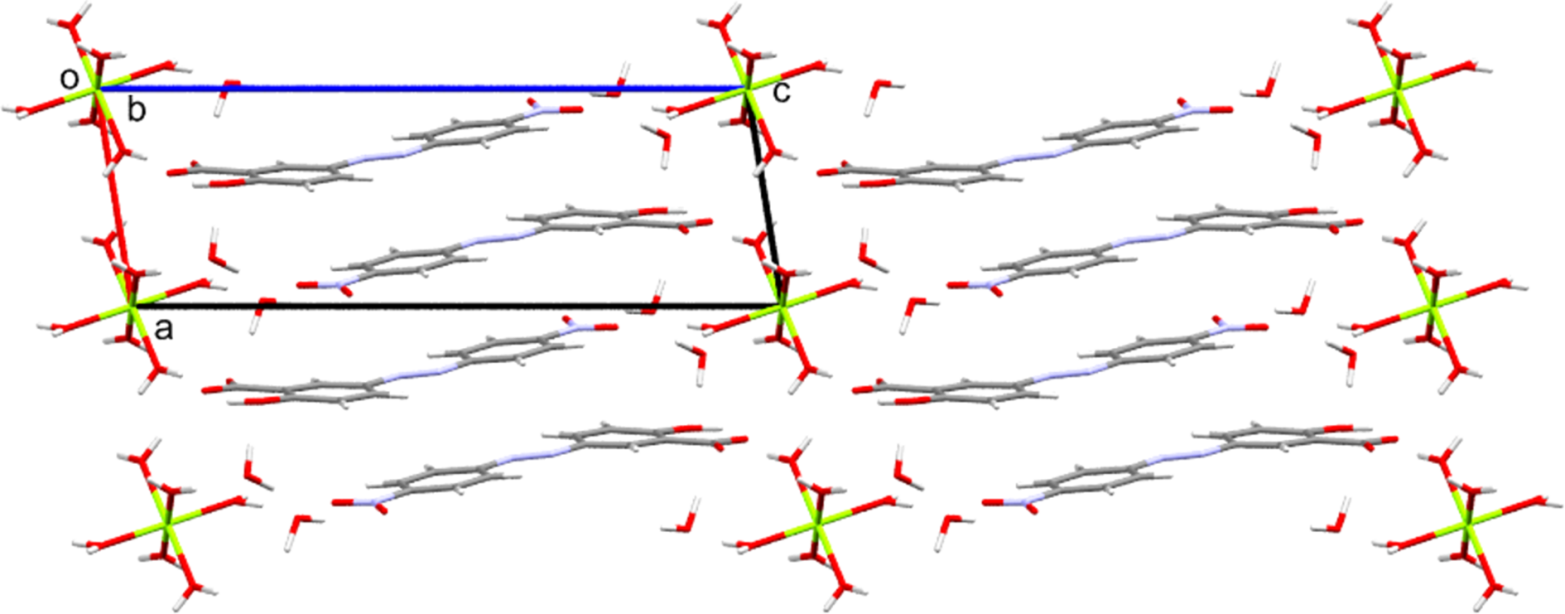
Packing diagram of **MgMO**, viewed along the *b*-axis direction. Hydro­philic layers of Mg^II^ ions and water mol­ecules alternate with organic layers com­posed of the azo dye anions. All layers lie parallel to the crystallographic *ab* plane.

For Mg, this is the same SSIP structure type as was invariably found for sul­fon­ated azo dyes (Kennedy *et al.*, 2004 ▸; Kennedy *et al.*, 2012 ▸) and is a common though not exclusive motif for Mg salts of simple benzoate anions (Arlin *et al.*, 2011 ▸). However, only the Li salt of the di­sul­fon­ated dye Orange G has a similar [Li(H_2_O)_4_]^+^ cation, with other sul­fon­ated azo dyes forming Li-to-sulfonate bonds (Ojala *et al.*, 1994 ▸; Kennedy *et al.*, 2009 ▸). This suggests that **MO** is a poorer ligand for Li than are *para*- and *meta*-sul­fon­ated azo dyes (Kennedy *et al.*, 2006 ▸). The [Li(H_2_O)_4_]^+^ cation is also a rare counter-ion for *R*COO^−^ (*R* = ar­yl) species. As the crystallization solvents of many species are not readily apparent, care needs to be taken in inter­preting the numbers. However, even given that, it is remarkable that of the approximately 300 such Li/*R*COO^−^ structures available in the CSD, only one (refcode ZEGJUT; Quarez *et al.*, 2017 ▸) has only a simple [Li(H_2_O)_4_]^+^ cation. Both the **MgMO** and **LiMO** SSIP structures pack to give simple layered structures, with layers of organic anions alternating with hydro­philic layers of cations and water mol­ecules. The polar heads and tails of the dye anions inter­act with the hydro­philic layers *via* hy­dro­gen bonding to water (Figs. 3 ▸ and 4 ▸). Throughout all the metal salt structures herein, the only intermolecular classical hy­dro­gen-bond donors are the water H atoms. Of the potential hy­dro­gen-bond acceptors, most structural classes are utilized, with the notable exception of the azo N atoms, and so all hy­dro­gen bonds for the metal salts are of type O—H⋯O; see Table 8 ▸ for a summary.

**Figure 5:**
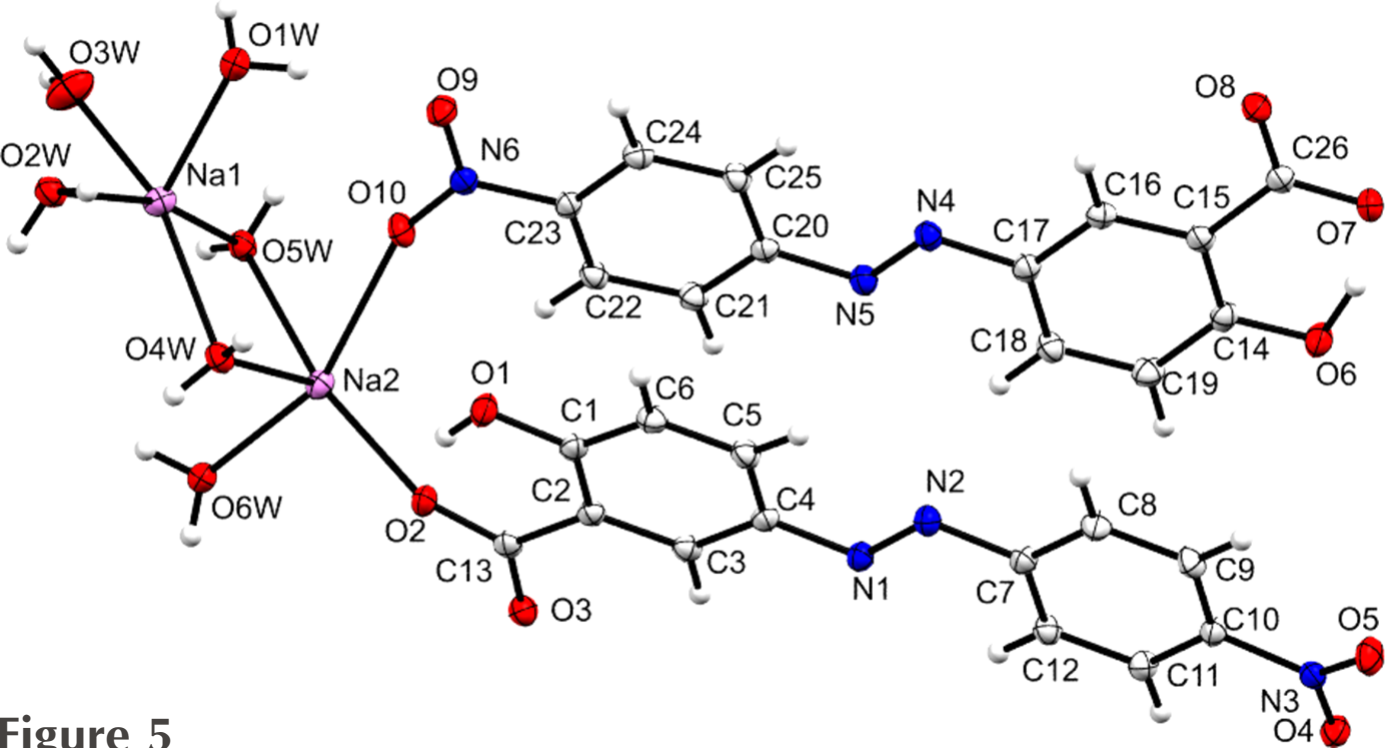
Contents of the asymmetric unit of **NaMO**.

Three structures contain heavier alkali metal ions (Figs. 5 ▸, 6 ▸ and 7 ▸). These are [Na(C_13_H_8_N_3_O_5_)(H_2_O)_3_], [Rb(C_13_H_8_N_3_O_5_)(H_2_O)_2_] and [CsNa(C_13_H_8_N_3_O_5_)_2_(H_2_O)_6_], hereafter **NaMO**, **RbMO** and **CsNaMO**, respectively. No other bimetallic Na/Cs benzoate-derivative structures are known, but the di­sulfonate azo dye Orange G does form a mixed Na/Cs salt (Kennedy *et al.*, 2006 ▸). Note that none of the seven **MO** structures reported herein feature metal bonding to either the OH or N=N groups. However, an inter­esting structural feature is that all three of the heavier alkali metal structures have both metal-to-carboxyl­ate and metal-to-nitro bonds. It has been shown previously for simple nitro­benzoate anions that nitro groups are com­petitive with formally charged carboxyl­ate groups with respect to their ability to bind to Group 1 metals in the solid state (*e.g.* Smith, 2015 ▸). For all three alkali metals, the shortest *M*—O bond is always to a carboxyl­ate O atom, but for both Rb and Cs, the ranges of the longer *M*—O(carboxyl­ate) bonds and of the *M*—O(nitro) bonds overlap (see Tables 3 ▸–5 ▸ ▸).

**Figure 8:**
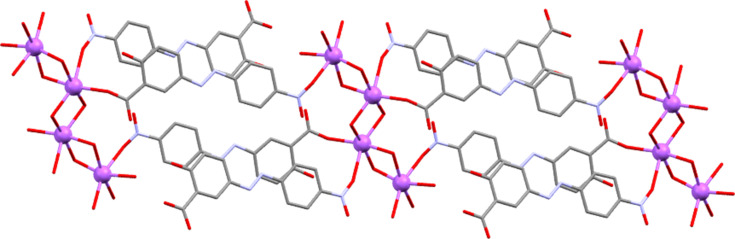
Part of the 1D coordination polymer of **NaMO**, highlighting the core Na_4_ units that are linked through the length of the azo anions by head and tail bonding to –COO^−^ and –NO_2_ groups. H atoms have been omitted for clarity.

The asymmetric unit of **NaMO** contains two Na centres, two azo anions and six water ligands. Of the latter, three bridge between two Na centres and three are terminal. Both crystallographically independent Na centres are six-coordinate, but Na1 bonds to all three of the terminal water ligands, whilst the only non-bridging inter­action for Na2 is a single bond to nitro atom O10. As Na1 makes relatively few bridged inter­actions, an extended structure based on Na_4_ motifs results (see Fig. 8 ▸). Each Na_4_ unit has two bridging water mol­ecules between each Na pair, with the terminal ligands on Na1 and Na1^ii^ [symmetry code: (ii) −*x* + 2, −*y* + 1, −*z*] capping the fragment and ending its propagation. Each Na_4_ unit then bridges to two neighbouring Na_4_ units, with each bridge utilizing head and tail, carboxylate and nitro, bonding through two azo anions. This through dye linkage creates a 1D coordination polymer of linked Na_4_ units that propagates parallel to the crystallographic *bc* diagonal. It also results in a similar alternating organic/inorganic layered structure to those seen for the Li and Mg SSIP species above.

**Figure 9:**
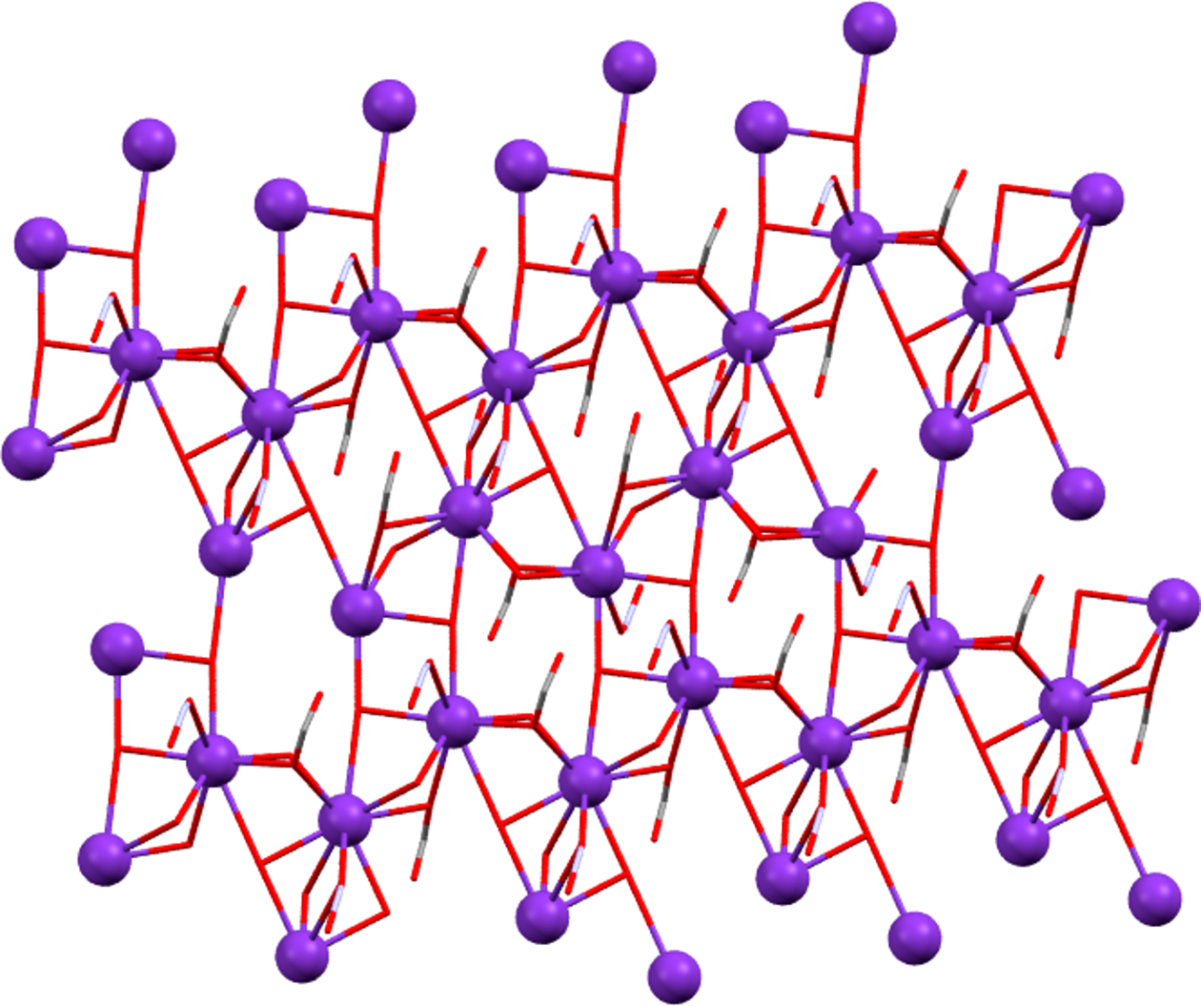
The 2D network formed *via* Rb—O—Rb bridges in the structure of **RbMO**.

Like **NaMO**, the asymmetric unit of **RbMO** contains two Rb centres and two azo anions, but only four water ligands. A crucial difference from **NaMO** is that in **RbMO** all ligands, both azo and water, have bridging roles. Together with the higher coordination numbers of the metal centres, this leads to a coordination polymer that is 3D rather than 1D. Rb—O—Rb bridges (where O is from a water, nitro or carboxyl­ate group) and, in the cases of O1*W* and O3*W*, O atoms bridging three Rb centres give the 2D net shown in Fig. 9 ▸. Both crystallographically independent azo anions utilize both their COO^−^ and NO_2_ groups to bind to and bridge between these layers, giving the overall 3D coordination polymer. This, once again, gives an overall simple layering structure, as seen above.

**Figure 10:**
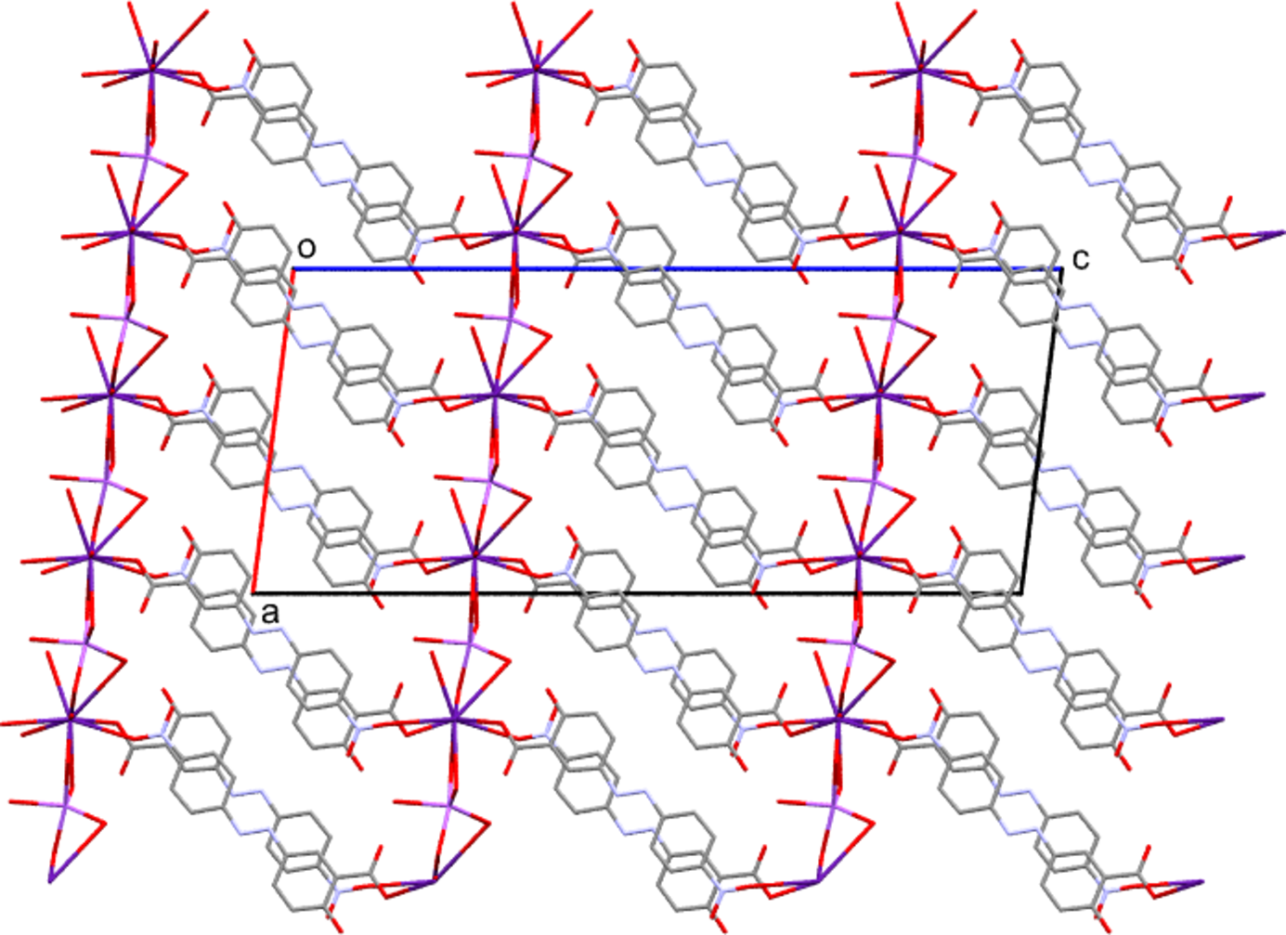
View of the packing in **CsNaMO** looking down the crystallographic *b* direction. Inorganic and organic layers lie parallel to *ab*. H atoms have been omitted for clarity.

The asymmetric unit of **CsNaMO** contains a Cs centre, a Na centre, two azo anions and six water ligands. Each metal has a single terminal water ligand, but the other four waters bridge between metal centres. As with **RbMO**, a 2D layer is formed by *M*—O—*M* bridges, but here all the O-atom bridges are from water ligands. The 2D layers are connected to each other through the length of an azo anion *via* bonding to both the COO^−^ and NO_2_ groups, but as the Na centre does not bond to the azo anion, this solely connects Cs to Cs. As with all structures discussed so far, the overall packing is a simple layered structure (Fig. 10 ▸).

The two heavy Group 2 metal salts isolated are [Sr(C_13_H_8_N_3_O_5_)_2_(H_2_O)_2_(DMF)]·2DMF and [Ba(C_13_H_8_N_3_O_5_)_2_(H_2_O)_2_(DMF)]·2DMF, denoted **SrMO** and **BaMO**, respectively. As indicated by the unit-cell and symmetry data given in Table 1 ▸, the two structures are isostructural and isomorphous. The larger Ba^2+^ ion causes the **BaMO** unit cell to be slightly larger than that of **SrMO** (3.1% increase), but this expansion is anisotropic, with *a* and *b* increasing (by 3.7 and 0.9%, respectively), while *c* decreases (by 1.3%). The *a* direction is also the direction of growth of the Sr—O—Sr-based 1D coordination polymer and thus the main direction of unit-cell expansion can simply be attributed to the difference in bond length between Sr—O and Ba—O. Similar effects have been seen for other isostructural Sr/Ba com­plexes with benzoate based ligands (Allan *et al.*, 2018 ▸).

**Figure 11:**
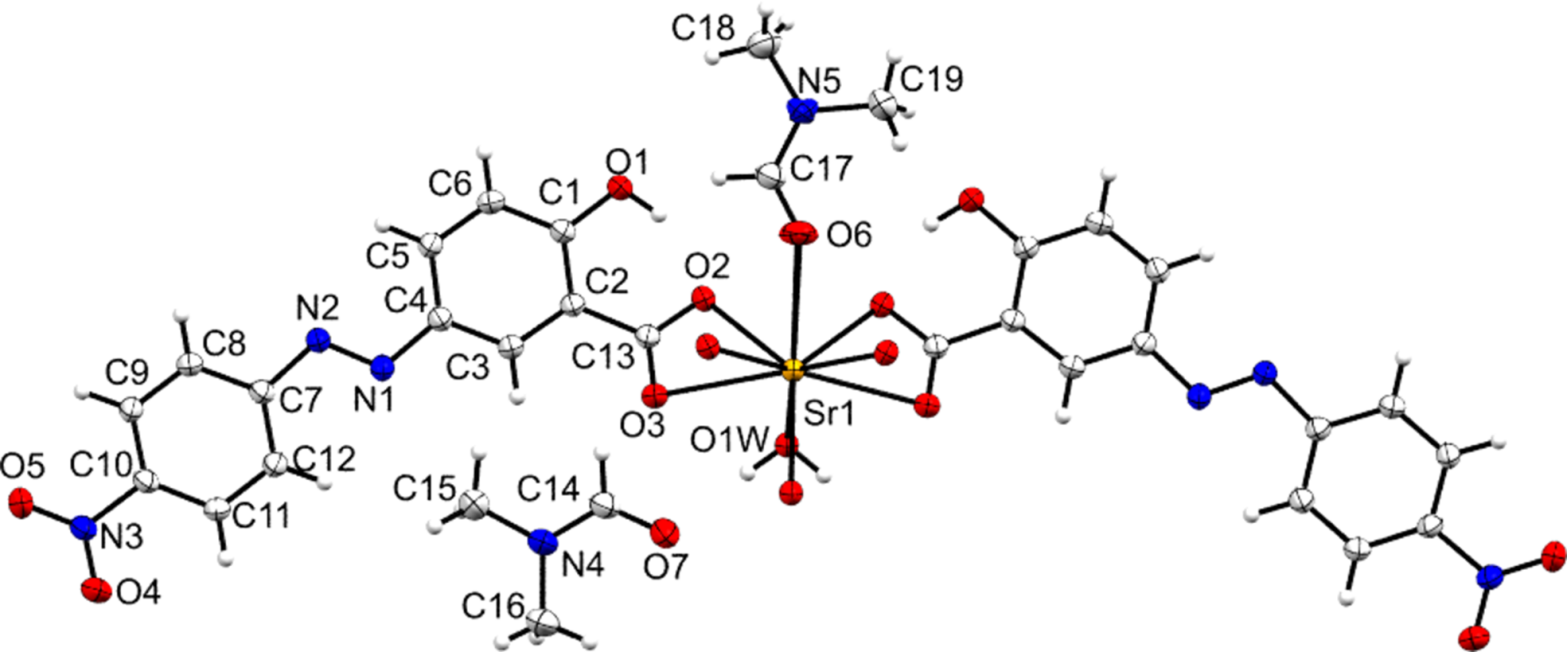
The asymmetric unit contents of **SrMO** expanded to show the complete coordination of the metal centre. Unlabelled atoms are generated by the action of the mirror plane at *y* = 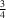. The disordered equivalent of the DMF ligand has been omitted for clarity. The three O atoms shown as bound solely to Sr1 are symmetry equivalents of O1*W* and O2. All bridges between Sr centres involve these atoms (see Table 6 ▸).

**Figure 13:**
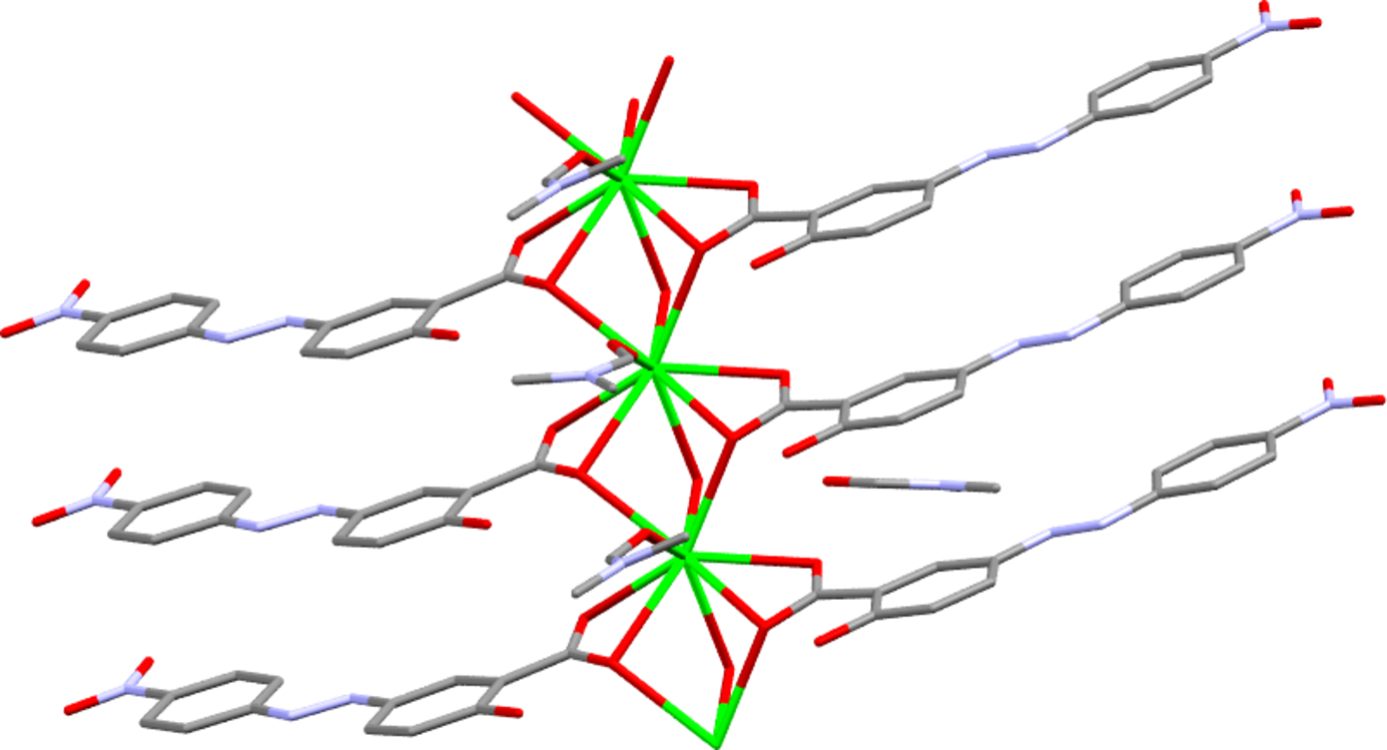
Part of the 1D coordination polymer of **SrMO**, with H atoms omitted for clarity. The coordination polymer propagates in the crystallographic *a* direction. The **BaMO** structure is isostructural.

In both structures, a crystallographic mirror plane passes through the metal centre, the O1*W* water ligand and the DMF ligand. This causes the latter to be disordered. The asymmetric unit of each thus contains half a metal centre, an azo anion, half of a water ligand, half of a DMF ligand and a DMF solvent mol­ecule (*Z*′ = 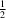) (Figs. 11 ▸ and 12 ▸). Unlike the heavier Group 1 metal structures, there are no metal-to-nitro bonds in either structure, although bonding to nitro does have precedent for Group 2 metals with nitro­benzoate anions (Arlin *et al.*, 2011 ▸). This leaves the water ligand and the COO^−^ groups to provide *M*—O—*M* bridges, with the result being 1D coordination polymers (see Fig. 13 ▸). Tables 6 ▸ and 7 ▸ give the *M*—O bond lengths for **SrMO** and **BaMO**. Note that although these two structures are highly isostructural, there are small variations in detail with respect to bonding. Thus, for instance, the longest bond in **SrMO** is an Sr-to-water bond, whilst in **BaMO**, the longest bond is from Ba to a carboxyl­ate O atom. The ability of isostructural structures to tolerate similar small changes within an otherwise shared packing motif has been described (Bombicz, 2024 ▸; Kennedy *et al.*, 2024 ▸).

**Figure 14:**
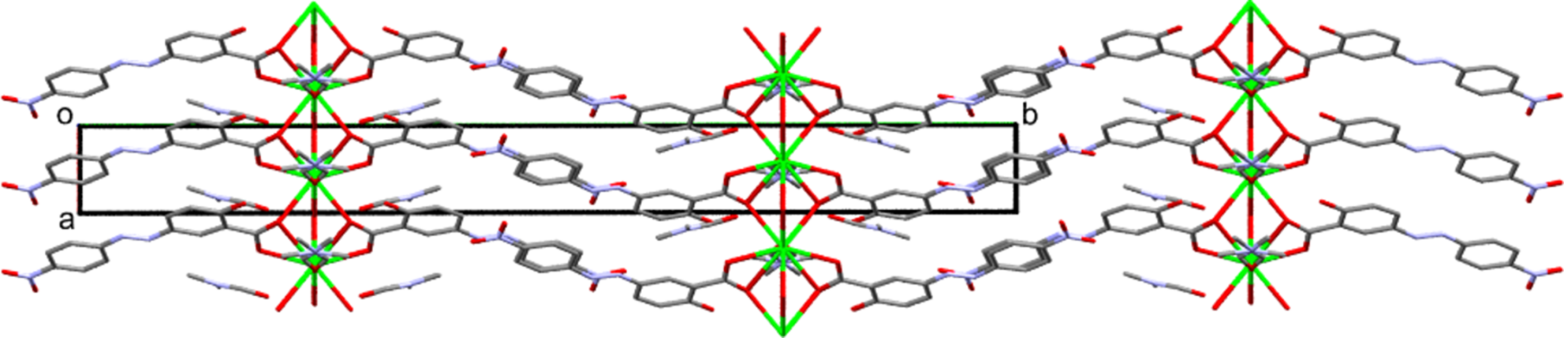
Packing in **SrMO**, viewed along the *c*-axis direction. H atoms have been omitted for clarity.

Differences in the ligating behaviour of the azo anions and in inter­molecular inter­action types lead to **SrMO** and **BaMO** having different layering structures to the other salt forms discussed. All of the SSIP and Group 1 metal structures of **MO** have simple layered structures with the azo anions bridging between the inorganic/polar layers and utilizing both their salicylate and NO_2_ functional groups to bond to these layers either by direct *M*—O bonds or by hy­dro­gen bonding to the water atoms present. Not only do the nitro groups in **SrMO** and **BaMO** not form *M*—O bonds, they also do not act as hy­dro­gen-bond acceptors and thus do not inter­act in any way with the metal centres or the associated solvent mol­ecules; see Table 8 ▸ for hy­dro­gen-bonding summary. A further difference is that, whilst in all structures herein the azo anions inter­act with each other through π-stacking-type inter­actions, in **SrMO** and **BaMO** these stacks involve parallel azo anions, whilst in all other structures the azo anions are anti­parallel, with nitro­benzene rings inter­acting with salicylate rings. The packing of **SrMO** and **BaMO** is thus different from all the others, as in these structures the azo ions do not form simple links between inorganic layers. Instead, nitro­benzene groups lie in the centre of an organic bilayer (see Fig. 14 ▸) and the organic arms of the 1D polymers inter­digitate with each other.

**Figure 15:**
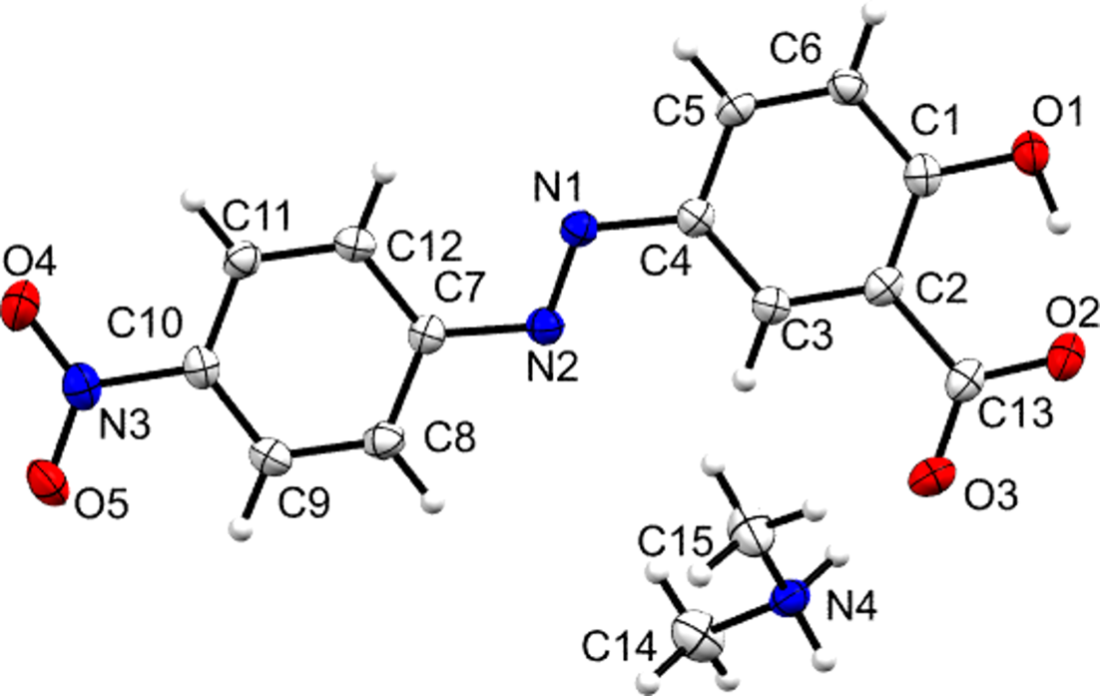
The asymmetric unit contents of **[NMe_2_H_2_]MO**.

The final structure pre­sent­ed herein is that of [NH_2_Me_2_]^+^[C_13_H_8_N_3_O_5_]^−^, **[NMe_2_H_2_]MO** (Fig. 15 ▸). Di­methyl­amine is a known degradation product of DMF (Comins & Joseph, 2001 ▸) and as **[NMe_2_H_2_]MO** was isolated from an aged solution of **NaMO** in DMF, this seems a likely source of the cation. Ammonium, NH_4_^+^, salts of sul­fon­ated azo dyes are known to often be isostructual with the equivalent K^+^ or Rb^+^ salt form (Kennedy *et al.*, 2024 ▸), but little relevant solid-state structural work is known for larger methyl­ammonium cations, although the structure of the NMe_4_^+^ salt of **MO** has been reported (Yatsenko & Paseshnichenko, 2016 ▸). The main hy­dro­gen-bonding motif observed in **[NMe_2_H_2_]MO** utilizes both of the cation H atoms as donors and carboxyl­ate atom O3 as an acceptor to form a 1D 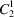
(4) chain that is generated by the 2_1_ axis of the crystal.

All the azo anions in this study have very similar conformations and geometries. All have two essentially coplanar aromatic rings {the maximum dihedral angle between ring planes is 9.52 (7)° for **[NMe_2_H_2_]MO**} and all the COO^−^ and NO_2_ functionalities are also coplanar with their parent rings. In the case of the carboxyl­ate groups, this planarity is favoured by a ubiquitous intra­molecular hy­dro­gen bond between the –OH and –COO^−^ groups. The details of the bond geometry are also similar throughout the group. Concentrating on the main atoms of the chromophore, the N=N distance varies from 1.248 (8) to 1.275 (7) Å (for **BaMO** and **CsNaMO**, respectively), whilst the C—N distance ranges are 1.411 (2)–1.426 (3) and 1.421 (8)–1.433 (3) Å for the salicylate- and nitro-substituted rings, respectively (distances for structures **SrMO**, **LiMO**, **CsNaMO** and **LiMO**). The com­parable bond lengths for the free acid structure (1.250, 1.421 and 1.426 Å) sit in the middle of these ranges (Yatsenko & Paseshnichenko, 2014 ▸). A major contrast to the sul­fon­ated azo dyes is thus that the **MO** free acid is protonated at the COO^−^ function – and that makes no difference to the geometry of the azo chromophore or to its colour as com­pared to anionic forms. In the absence of basic side groups, sul­fon­ated azo dyes protonate at the N=N group, leading typically to large geometric differences in the chromophore and to a substantial colour change at low pH. **MO** is used as a pH indicator, but solutions transform from yellow to red only at approximately pH 12. This fits with the loss of the phenol OH proton rather than with any change at the COO^−^ group.

## Summary

The structures of *s*-block metal salt forms of **MO** show many structural features similar to those found for the more well-investigated *s*-block metal salt forms of sul­fon­ated azo dyes. Thus, for instance, they all form layered structures and the Mg form **MgMO** is an SSIP with an [Mg(H_2_O)_6_]^2+^ cation. How­ever, there are also some inter­esting differences. **LiMO** also forms an SSIP. For sul­fon­ated monoazo dyes, only Orange G has a similar SSIP structure for Li, perhaps indicating that **MO** is a poorer ligand for *s*-block metals than most sul­fon­ated monoazo dyes (Kennedy *et al.*, 2004 ▸; Kennedy *et al.*, 2006 ▸; Kennedy *et al.*, 2012 ▸). The Sr and Ba salt forms of *para*- or *meta*-sul­fon­ated monoazo dyes were classified as ‘Class 2, simple com­plexes’ where metal-to-SO_3_ bonding was present and water ligands were exclusively terminal (Kennedy *et al.*, 2004 ▸; Kennedy *et al.*, 2009 ▸; Kennedy *et al.*, 2012 ▸). This led to simple mono- or dimeric metal com­plexes. In contrast, **SrMO** and **BaMO** are both relatively com­plex 1D coordination polymers, and both feature *M*—O—*M* units where the water mol­ecule is bridging. Thus, despite having a simpler planar –COO^−^ substituent over a tetra­hedral SO_3_ substituent, and despite **LiMO** indicating less inclination for **MO** to bond to *s*-block metals, more com­plex species with more azo-to-metal coordination arise for **MO** here. Finally, the Na salts of sul­fon­ated azo dyes, along with the heavier Group 1 metal salts, were categorized as ‘Class 3, higher connectivity com­plexes’. These were typically 2D or 3D coordination polymers and all featured bridging water ligands. **NaMO** would initially seem to fit this description, albeit with a somewhat lower than normal 1D coordination polymer. However, the previous classifications only considered coordination of water and of the sulfonate group. Any links formed *via* other substituents on the azo anions were ignored. If this formal stipulation is observed, then **NaMO** becomes a discrete Na_4_ unit and fits poorly with the previously described structural units.

## Supplementary Material

Crystal structure: contains datablock(s) LiMO, NaMO, RbMO, CsNaMO, MgMO, SrMO, BaMO, NMe2H2MO, global. DOI: 10.1107/S2053229626006765/jx3107sup1.cif

Structure factors: contains datablock(s) LiMO. DOI: 10.1107/S2053229626006765/jx3107LiMOsup2.hkl

Structure factors: contains datablock(s) NaMO. DOI: 10.1107/S2053229626006765/jx3107NaMOsup3.hkl

Structure factors: contains datablock(s) RbMO. DOI: 10.1107/S2053229626006765/jx3107RbMOsup4.hkl

Structure factors: contains datablock(s) CsNaMO. DOI: 10.1107/S2053229626006765/jx3107CsNaMOsup5.hkl

Structure factors: contains datablock(s) MgMO. DOI: 10.1107/S2053229626006765/jx3107MgMOsup6.hkl

Structure factors: contains datablock(s) SrMO. DOI: 10.1107/S2053229626006765/jx3107SrMOsup7.hkl

Structure factors: contains datablock(s) BaMO. DOI: 10.1107/S2053229626006765/jx3107BaMOsup8.hkl

Structure factors: contains datablock(s) NMe2H2MO. DOI: 10.1107/S2053229626006765/jx3107NMe2H2MOsup9.hkl

CCDC references: 2565190, 2565189, 2565188, 2565187, 2565186, 2565185, 2565184, 2565183

## Figures and Tables

**Figure 2:**
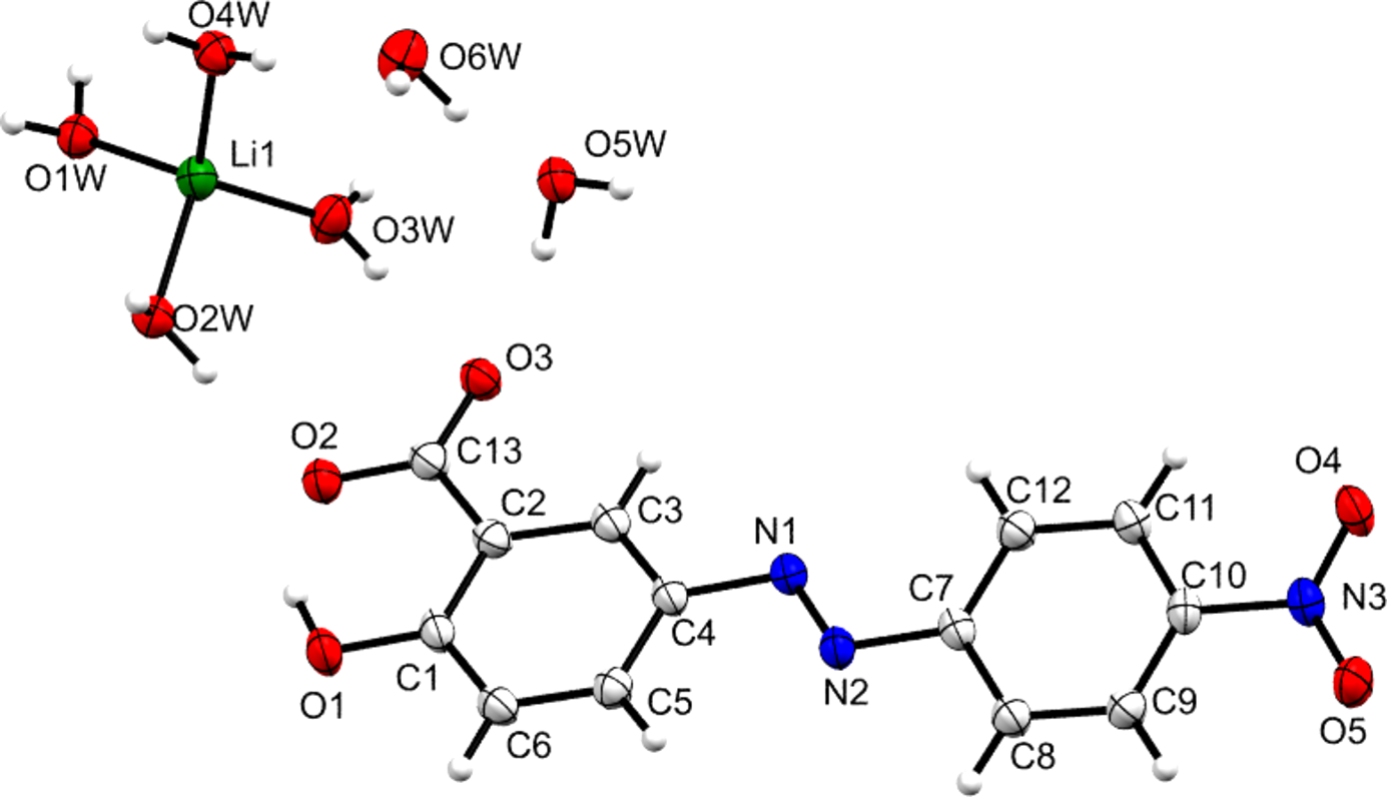
Contents of the asymmetric unit of **LiMO**.

**Figure 4:**
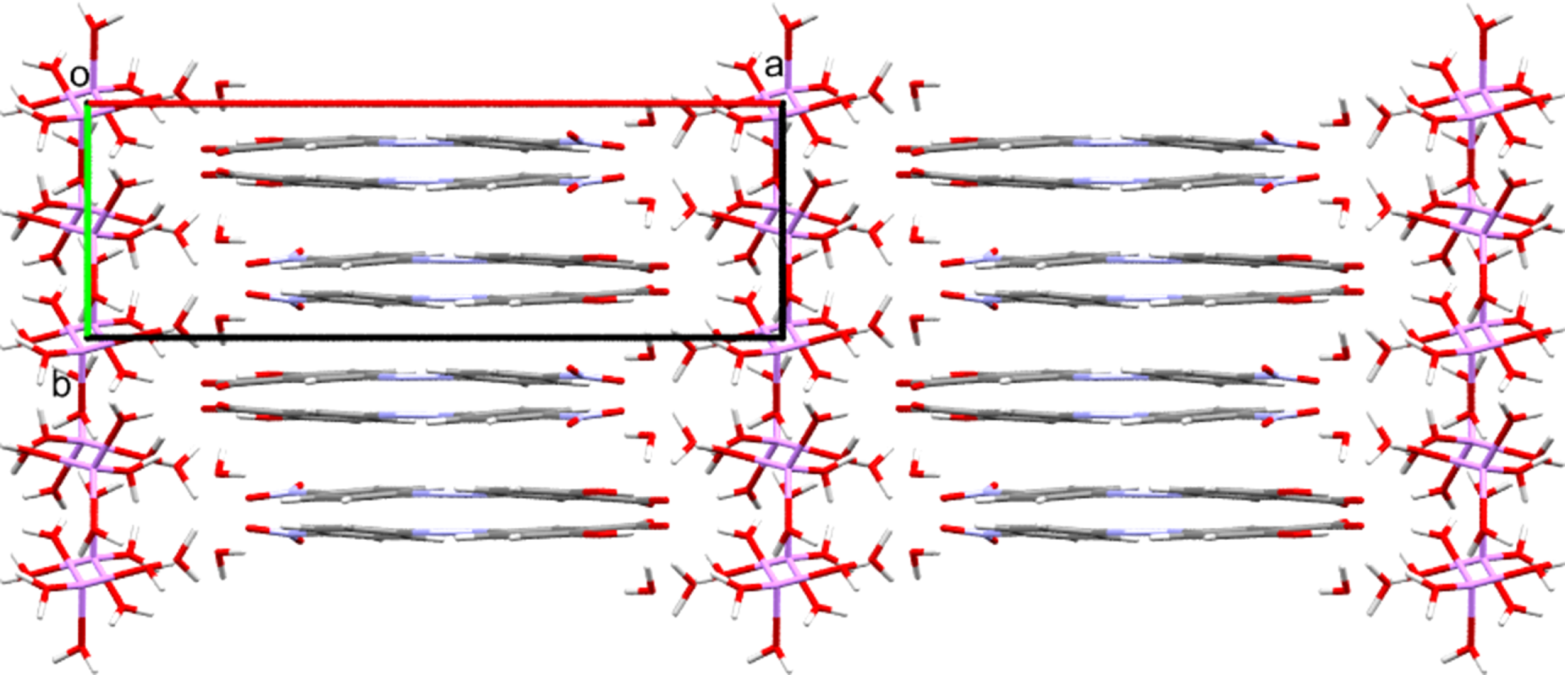
Packing diagram of **LiMO**, viewed along the *c*-axis direction. Hydro­philic layers of Li^I^ ions and water mol­ecules alternate with organic layers com­posed of the azo dye anions. All layers lie parallel to the crystallographic *bc* plane.

**Figure 6:**
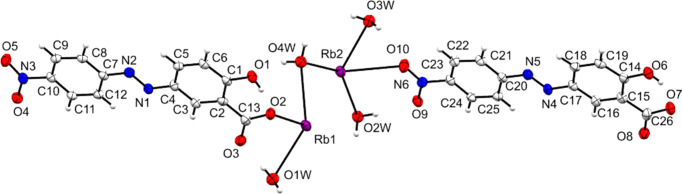
Contents of the asymmetric unit of **RbMO**.

**Figure 7:**
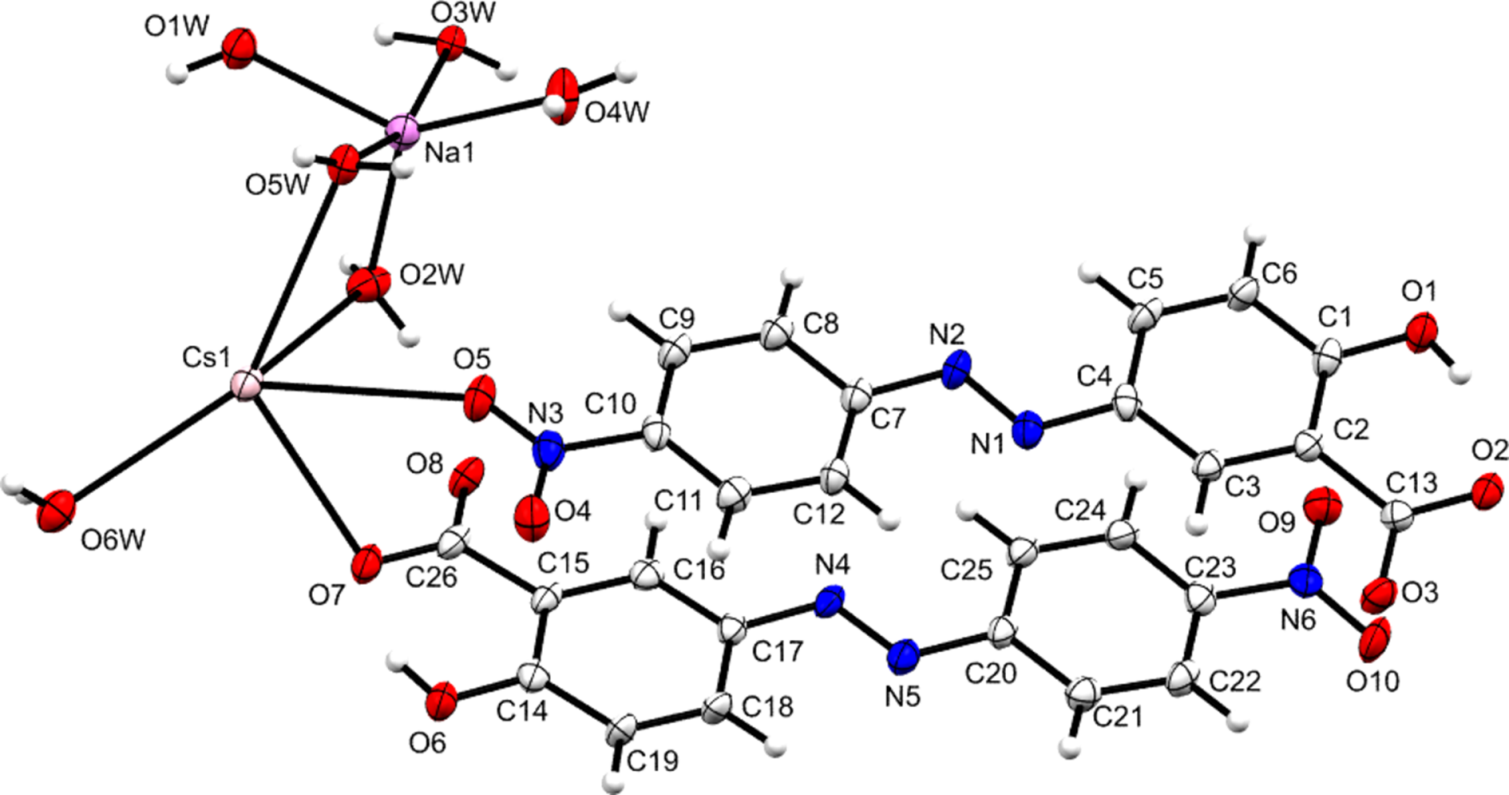
Contents of the asymmetric unit of **CsNaMO**. Note that O7*W*, the minor-occupancy disordered site for the majority occupancy O3*W*, has been omitted for clarity.

**Figure 12:**
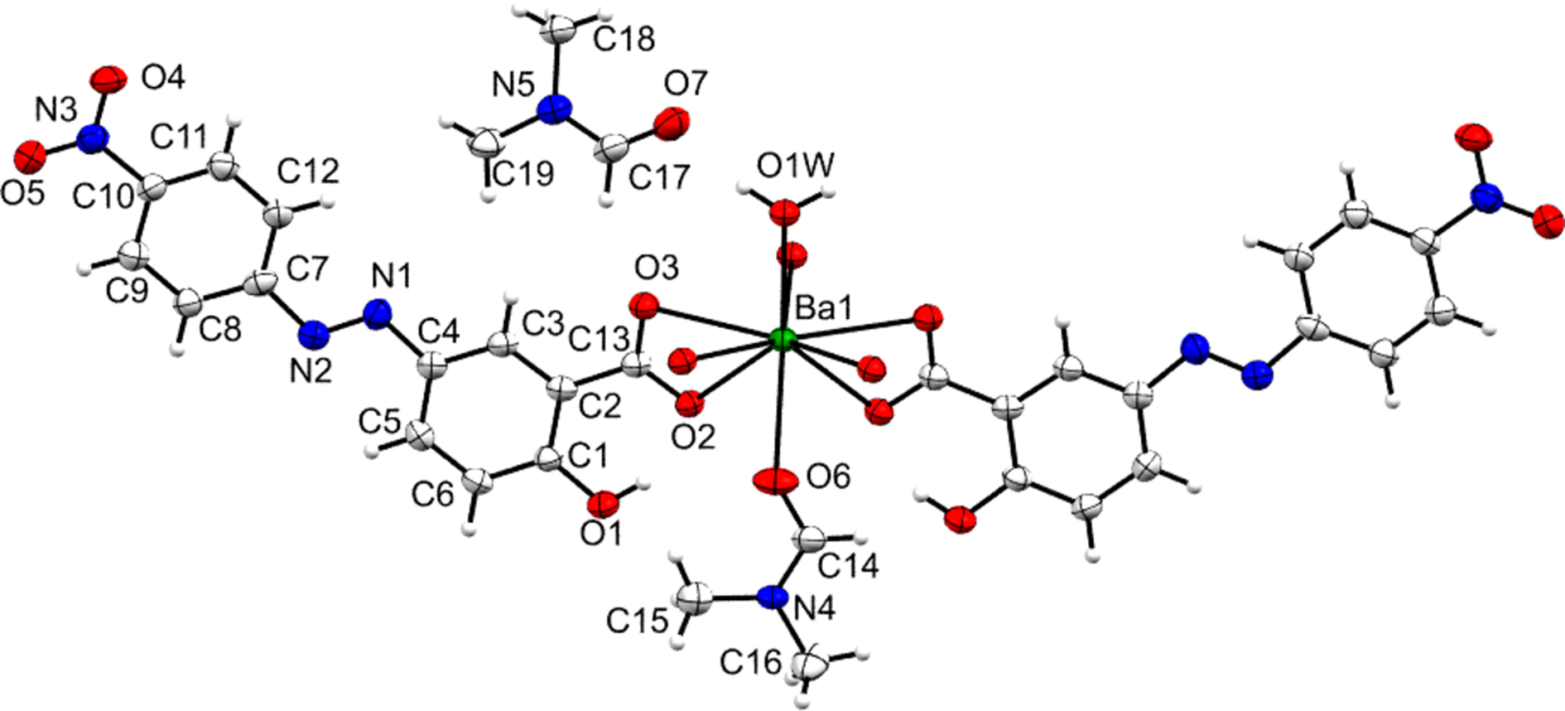
The asymmetric unit contents of **BaMO** expanded to show the complete coordination of the metal centre. Unlabelled atoms are generated by the action of the mirror plane at *y* = 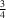. The disordered equivalent of the DMF ligand has been omitted for clarity. As with the isostructural **SrMo** shown in Fig. 11 ▸, bridging to other Ba centres is *via* O2 and O1*W* (see Table 7 ▸ for details).

**Table:** Experiments were carried out with Cu *K*α radiation using a Rigaku Synergy-i diffractometer. H atoms were treated by a mixture of independent and constrained refinement. Absorption was corrected for by multi-scan methods (*CrysAlis PRO*; Rigaku OD, 2019 ▸).

	**LiMO**	**NaMO**	**RbMO**	**CsNaMO**
Crystal data				
Chemical formula	[Li(H_2_O)_4_](C_13_H_8_N_3_O_5_)·2H_2_O	[Na(C_13_H_8_N_3_O_5_)(H_2_O)_3_]	[Rb(C_13_H_8_N_3_O_5_)(H_2_O)_2_]	[NaCs(C_13_H_8_N_3_O_5_)_2_(H_2_O)_6_]
*M*_r_	401.26	363.26	407.73	836.44
Crystal system, space group	Monoclinic, *P*2_1_/*c*	Triclinic, *P* 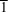	Triclinic, *P* 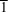	Monoclinic, *Ia*
Temperature (K)	100	100	100	100
*a*, *b*, *c* (Å)	20.6053 (8), 6.6518 (2), 13.9151 (6)	7.0932 (1), 12.9590 (2), 17.0280 (2)	7.2937 (3), 12.3381 (5), 16.6814 (10)	14.4842 (1), 6.6401 (1), 34.0053 (3)
α, β, γ (°)	90, 106.597 (4), 90	77.218 (1), 81.058 (1), 83.777 (1)	81.556 (4), 84.029 (4), 89.019 (3)	90, 97.156 (1), 90
*V* (Å^3^)	1827.78 (12)	1503.43 (4)	1476.83 (12)	3245.04 (6)
*Z*	4	4	4	4
μ (mm^−1^)	1.11	1.40	5.04	9.79
Crystal size (mm)	0.40 × 0.05 × 0.01	0.28 × 0.08 × 0.05	0.19 × 0.17 × 0.03	0.34 × 0.05 × 0.03

Data collection				
*T*_min_, *T*_max_	0.685, 1.000	0.586, 1.000	0.742, 1.000	0.430, 1.000
No. of measured, independent and observed [*I* > 2σ(*I*)] reflections	9245, 3317, 2461	30571, 5799, 5422	10735, 10735, 9496	65752, 6112, 6036
*R*_int_	0.049	0.033	0.060	0.074
(sin θ/λ)_max_ (Å^−1^)	0.605	0.615	0.616	0.615

Refinement				
*R*[*F*^2^ > 2σ(*F*^2^)], *wR*(*F*^2^), *S*	0.058, 0.176, 1.06	0.031, 0.088, 1.03	0.056, 0.171, 1.15	0.037, 0.094, 1.04
No. of reflections	3317	5799	10735	6112
No. of parameters	305	507	474	492
No. of restraints	1	2	12	20
Δρ_max_, Δρ_min_ (e Å^−3^)	0.30, −0.36	0.24, −0.33	1.39, −1.60	1.73, −0.86
Absolute structure	–	–	–	Refined as an inversion twin
Absolute structure parameter	–	–	–	0.492 (6)

**Table:** 

	**MgMO**	**SrMO**	**BaMO**	**NMe_2_H_2_MO**
Crystal data				
Chemical formula	[Mg(H_2_O)_6_](C_13_H_8_N_3_O_5_)_2_·4H_2_O	[Sr(C_13_H_8_N_3_O_5_)_2_(C_3_H_7_NO)(H_2_O)]·2C_3_H_7_NO	[Ba(C_13_H_8_N_3_O_5_)_2_(C_3_H_7_NO)(H_2_O)]·2C_3_H_7_NO	C_2_H_8_N^+^·C_13_H_8_N_3_O_5_^−^
*M*_r_	776.92	897.37	947.09	332.32
Crystal system, space group	Triclinic, *P* 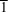	Monoclinic, *P*2_1_/*m*	Monoclinic, *P*2_1_/*m*	Monoclinic, *P*2_1_/*n*
Temperature (K)	100	100	100	120
*a*, *b*, *c* (Å)	6.7321 (2), 6.8541 (2), 19.2031 (6)	3.9057 (1), 42.0234 (6), 11.4398 (1)	4.0516 (1), 42.4029 (12), 11.2890 (4)	6.1197 (1), 7.8725 (2), 31.5876 (8)
α, β, γ (°)	85.401 (3), 79.929 (3), 74.380 (3)	90, 94.490 (1), 90	90, 95.425 (4), 90	90, 90.049 (2), 90
*V* (Å^3^)	839.69 (5)	1871.86 (6)	1930.76 (10)	1521.81 (6)
*Z*	1	2	2	4
μ (mm^−1^)	1.32	2.72	8.70	0.94
Crystal size (mm)	0.22 × 0.15 × 0.11	0.22 × 0.12 × 0.06	0.37 × 0.04 × 0.03	0.25 × 0.20 × 0.15

Data collection				
*T*_min_, *T*_max_	0.910, 1.000	0.592, 1.000	0.631, 1.000	0.659, 1.000
No. of measured, independent and observed [*I* > 2σ(*I*)] reflections	7561, 3224, 2506	19910, 3661, 3409	17995, 3734, 3457	12393, 2944, 2469
*R*_int_	0.032	0.043	0.067	0.033
(sin θ/λ)_max_ (Å^−1^)	0.615	0.615	0.615	0.614

Refinement				
*R*[*F*^2^ > 2σ(*F*^2^)], *wR*(*F*^2^), *S*	0.057, 0.179, 1.08	0.030, 0.078, 1.05	0.061, 0.156, 1.10	0.047, 0.116, 1.08
No. of reflections	3224	3661	3734	2944
No. of parameters	285	304	299	231
No. of restraints	16	5	3	0
Δρ_max_, Δρ_min_ (e Å^−3^)	0.91, −0.33	0.43, −0.60	2.85, −1.44	0.34, −0.20

Computer programs: *CrysAlis PRO* (Rigaku OD, 2019 ▸), *SHELXT* (Sheldrick, 2015*a* ▸), *SHELXL2018* (Sheldrick, 2015*b* ▸), *Mercury* (Macrae *et al.*, 2020 ▸) and *SHELXL* in *WinGX* (Farrugia, 2012 ▸).

**Table 2:** Selected information on metal coordination ‘SSIP’ is solvent-separated ion pair, ‘CP’ is coordination polymer and *M*-to-O contact is defined as a bond using default radius values within *SHELXL*

Metal ion (*M*)	Coordination number	Bonds to –COO^−^	Bonds to –NO_2_	Bonds to water	Bonds to DMF	Structure type
Li	4	0	0	4	0	SSIP
Na1	6	0	1	5	0	1D CP
Na2	6	1	1	4	0	
Rb1	10	3	2	5	0	3D CP
Rb2	9	3	1	5	0	
Cs	9	2	2	5	0	3D CP
Na	5	0	0	5	0	
Mg	6	0	0	6	0	SSIP
Sr	9	6	0	2	1	1D CP
Ba	9	6	0	2	1	1D CP

**Table 3:** Selected bond lengths (Å) for **NaMO**

Na1—O1*W*	2.3722 (11)	Na2—O5*W*	2.3496 (10)
Na1—O3*W*	2.2957 (11)	Na2—O6*W*	2.4036 (10)
Na1—O4*W*	2.3930 (10)	Na2—O6*W*^ii^	2.4295 (10)
Na1—O5*W*	2.3953 (10)	Na2—O2	2.3536 (9)
Na1—O5^i^	2.4614 (10)	Na2—O10	2.6327 (10)
Na2—O4*W*	2.3468 (10)		

Symmetry codes: (i) 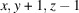; (ii) 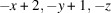.

**Table 4:** Selected bond lengths (Å) for **RbMO**

Rb1—O1*W*	3.016 (4)	Rb2—O1*W*^i^	3.406 (4)
Rb1—O1*W*^i^	2.909 (3)	Rb2—O2*W*	2.979 (3)
Rb1—O2*W*^ii^	2.918 (3)	Rb2—O3*W*^vii^	2.857 (3)
Rb1—O3*W*^iii^	3.349 (4)	Rb2—O3*W*	2.962 (4)
Rb1—O4*W*	3.108 (4)	Rb2—O4*W*	3.134 (4)
Rb1—O2	3.277 (4)	Rb2—O1^viii^	3.639 (4)
Rb1—O3	3.580 (4)	Rb2—O2^viii^	3.029 (3)
Rb1—O5^iv^	3.225 (4)	Rb2—O7^vi^	2.925 (4)
Rb1—O5^v^	3.276 (3)	Rb2—O10	3.058 (4)
Rb1—O7^vi^	2.985 (4)		

Symmetry codes: (i) 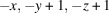; (ii) 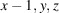; (iii) 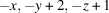; (iv) 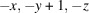; (v) 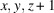; (vi) 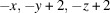; (vii) 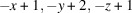; (viii) 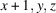.

**Table 5:** Selected bond lengths (Å) for **CsNaMO**

Cs1—O2*W*	3.183 (5)	Cs1—O7^iii^	3.092 (4)
Cs1—O3*W*^i^	3.111 (5)	Cs1—O10^ii^	3.177 (4)
Cs1—O4*W*^i^	3.659 (5)	Na1—O2*W*	2.309 (5)
Cs1—O5*W*	3.199 (4)	Na1—O3*W*	2.277 (5)
Cs1—O6*W*	3.147 (5)	Na1—O4*W*	2.325 (5)
Cs1—O2^ii^	3.264 (4)	Na1—O5*W*	2.295 (5)
Cs1—O5	3.169 (5)		

Symmetry codes: (i) 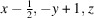; (ii) 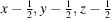; (iii) 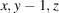.

**Table 6:** Selected bond lengths (Å) for **SrMO**

Sr1—O1*W*^i^	2.641 (2)	Sr1—O2	2.6946 (14)
Sr1—O1*W*	2.737 (2)	Sr1—O3^iii^	2.6368 (14)
Sr1—O2^i^	2.6384 (14)	Sr1—O3	2.6368 (14)
Sr1—O2^ii^	2.6384 (14)	Sr1—O6	2.559 (2)
Sr1—O2^iii^	2.6946 (14)		

Symmetry codes: (i) 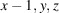; (ii) 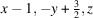; (iii) 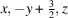.

**Table 7:** Selected bond lengths (Å) for **BaMO**

Ba1—O1*W*	2.819 (6)	Ba1—O2^iv^	2.848 (4)
Ba1—O1*W*^i^	2.846 (7)	Ba1—O3	2.793 (4)
Ba1—O2^ii^	2.781 (5)	Ba1—O3^iv^	2.793 (4)
Ba1—O2^iii^	2.781 (4)	Ba1—O6	2.669 (7)
Ba1—O2	2.848 (4)		

Symmetry codes: (i) 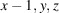; (ii) 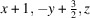; (iii) 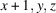; (iv) 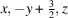.

**Table 8:** Showing which functional groups act as inter­molecular hy­dro­gen-bond acceptors; all hy­dro­gen-bond donors are water mol­ecules

	COO^−^	OH	NO_2_	N=N	Water	DMF
**LiMO**	5	0	1	0	6	n.a.
**NaMO**	6	1	2	0	2	n.a.
**RbMO**	5	1	1	0	1	n.a.
**CsNaMO**	5	1	2	0	2	n.a.
**MgMO**	5	1	2	0	4	n.a.
**SrMO**	0	0	0	0	0	1
**BaMO**	0	0	0	0	0	1
